# *Wls* Is Expressed in the Epidermis and Regulates Embryonic Hair Follicle Induction in Mice

**DOI:** 10.1371/journal.pone.0045904

**Published:** 2012-09-24

**Authors:** Sixia Huang, Xuming Zhu, Yanfang Liu, Yixin Tao, Guoyin Feng, Lin He, Xizhi Guo, Gang Ma

**Affiliations:** Bio-X Institutes, Key Laboratory for the Genetics of Developmental and Neuropsychiatric Disorders (Ministry of Education), Shanghai Jiao Tong University, Shanghai, People’s Republic of China; Ecole Normale Supérieure de Lyon, France

## Abstract

Wnt proteins are secreted molecules that play multiple roles during hair follicle development and postnatal hair cycling. Wntless (Wls) is a cargo protein required for the secretion of various Wnt ligands. However, its role during hair follicle development and hair cycling remains unclear. Here, we examined the expression of *Wls* during hair follicle induction and postnatal hair cycling. We also conditionally deleted *Wls* with K14-cre to investigate its role in hair follicle induction. *K14-cre;Wls^c/c^* mice exhibited abnormal hair follicle development, which is possibly caused by impaired canonical Wnt signaling. Meanwhile, *Wnt5a* is also expressed in embryonic epidermis, but *Wnt5a* null mice showed no significant defect in embryonic hair follicle morphogenesis. Therefore, *Wls* may regulate hair follicle induction by mediating the Wnt/β-catenin pathway.

## Introduction

Hair follicle development requires reciprocal signal crosstalks between the surface ectoderm and underlying mesenchyme [1]. In mice, hair follicle induction is initialized by a dermis-derived signal, which mediates the aggregation of epidermal keratinocytes to form placodes [2]. These placodes recruit specific fibroblasts from the mesenchyme to cluster underneath themselves and form dermal condensates that finally differentiate to dermal papillae. Subsequently, signals from both nascent placodes and dermal papillae coordinate the down-growth of placodes and the succeeding hair follicle morphogenesis [1]. Fully developed hair follicles undergo a cyclical process including growth (anagen), regression (catagen) and quiescence (telogen) [3].

Among several identified hair follicle inducting pathways [1], Wnt/β-catenin signaling is considered as the first epithelial signal that triggers placode formation [4]. Inhibiting epithelial Wnt activity by deleting β-catenin or overexpressing Dkk1, a Wnt inhibitor, will block placode formation [5], [6]. Although multiple Wnt ligands are redundantly expressed in the epithelium or underlying mesenchyme during hair follicle induction [7], only Wnt10b is characterized to mediate placode induction [4]. Whether other Wnts, especially non-canonical Wnts, also regulate hair follicle induction remains to be determined. Non-canonical Wnt members are reportedly expressed in embryonic skin and hair follicle compartments [7]. Among them, Wnt5a in dermal papillae is believed to act downstream of canonical Notch signaling in controlling postnatal hair follicle differentiation [8]. However, although Wnt5a begins to be expressed in the skin as early as E14.5, there is still no direct evidence clearly showing the role of Wnt5a in embryonic hair follicle development.

**Figure 1:**
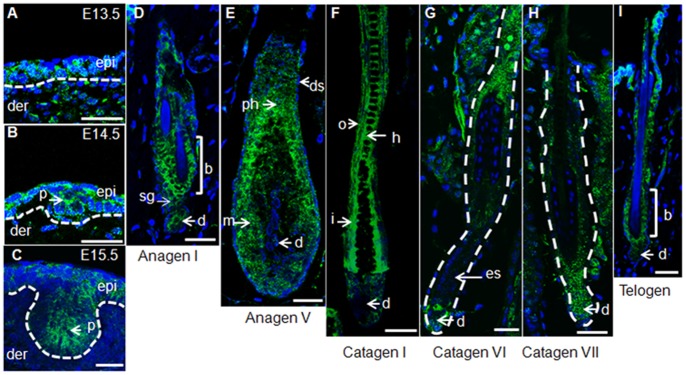
*Wls* is dynamically expressed during hair follicle induction and hair cycling. (A) Wls is expressed both in the surface ectoderm and underlying mesenchyme of the E13.5 embryo. (B) Wls expression is sustained in the epidermis and appears in nascent placodes at E14.5. (C) Wls is detected in the growing placodes and epidermis at E15.5. (D) *Wls* is expressed in the bulge region, second hair germ, and dermal papilla in early anagen. (E) Expression of *Wls* in full anagen. High-level *Wls* expression is observed in matrix keratinocytes and pre-hair shaft, the expression in dermal papilla becomes weak. The dermal sheath has no significant *Wls* expression. (F) Expression of *Wls* in early catagen. Wls is strongly expressed in ORS, IRS, and hair shaft, the expression in dermal papilla is increased. (G) Expression of Wls begins to decrease in mid-catagen, especially in epithelial strands. (H) Wls expression in late catagen mainly exists in lower hair follicles and the expression in dermal papilla is significant. (I) In telogen, Wls expression is mainly maintained in the bulge region, expression in dermal papilla is decreased. Bar = 30 µm. Abbreviations: b, bulge; d, dermal papilla; ds, dermal sheath; epi, epidermis; es, epithelial strand; h, hair shaft; i, inner root sheath; m, matrix; o, outer root sheath; ph, pre-hair shaft; sg, second hair germ.

Wntless is a trans-membrane protein that facilitates the secretion of various Wnt ligands from their producing cells [9], [10]. Wls function impairment causes developmental defects and diseases due to abnormal Wnt pathway activity. For example, total knockout Wls mice die early because of failure in embryonic body axis development similar to *Wnt3* null mice [11]. Wls is also required for the secretion of non-canonical Wnt5a in limb bud development [12]. In humans, Wls is involved in regulating bone mineral density and gliomatumourigenesis [13], [14]. Given that Wls is required for the secretion of almost all Wnts [15], taking advantage of *Wls* modification is a promising method for avoiding functional redundancy in determining the role of Wnts in hair follicle development.

In this study, we found that the expression of *Wls* in skin is dynamic at different stages during embryonic hair follicle development and postnatal hair cycling. To confirm whether Wls is required for hair follicle induction, we also deleted *Wls* by epidermal K14-cre. *K14-cre;Wls^c/c^* mice displayed defective hair follicle induction similar to *K14-cre;β-catenin^c/c^* mice. We also found significant Wnt5a expression in embryonic epidermis, indicating the potential role of non-canonical Wnts in hair follicle induction. We speculated the possible role of Wnt5a to hair follicle initiation may contribute to the phenotype of *K14-cre;Wls^c/c^* mice. However, no obvious defect of placode formation in *Wnt5a* deficient skin was observed, and the subsequent down-growth of placodes also seems normal in this mutant. Thus, although Wnt5a is expressed in epidermis and Wls is required for the secretion of Wnt5a, Wnt5a signaling in epidermis is not likely play an essential role in hair follicle induction. Our results suggest that Wls mediates the Wnt/β-catenin pathway to trigger hair follicle initiation.

**Figure 2:**
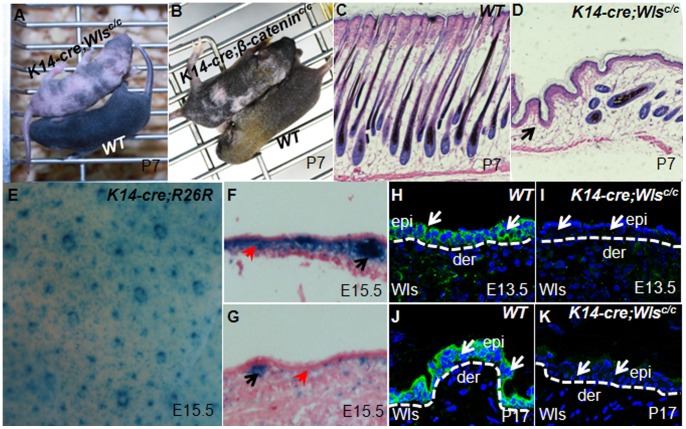
*K14-cre;Wls^c/c^* mice display patched hair loss. (A–B) Hairless patches are present in *K14-cre;Wls^c/c^* and *K14-cre;Wls^c/c^* mice. (C–D) Hair follicles disappear in hairless patches in *K14-cre;Wls^c/c^* mutant skin (the black arrow), whereas hair follicles are maintained in hairy patches but usually decrease in number and are progressively lost with aging. (E–G) K14-cre activity is not evenly distributed in the skin. Black arrows indicate placodes, and red arrows indicate inter-placode basal layers. (H–K) Wls protein is significantly reduced in the surface ectoderm at E13.5 and hairless patches at P17 in *K14-cre;Wls^c/c^* skin, indicating effective deletion of Wls by K14-cre. Abbreviations: epi, epidermis; der, dermis.

## Materials and Methods

### Mice

*Wls^c/c^*, *β-catenin^c/c^*, and *Wnt5a^+/−^* mice were prepared as previously described [12], [16]. K14-cre transgenic mice were provided by Dr. W. Birchmeier [5]. The ethical guidelines as well as other pertinent rules and regulations of Shanghai Jiao Tong University were followed strictly during all animal experiments.

**Figure 3:**
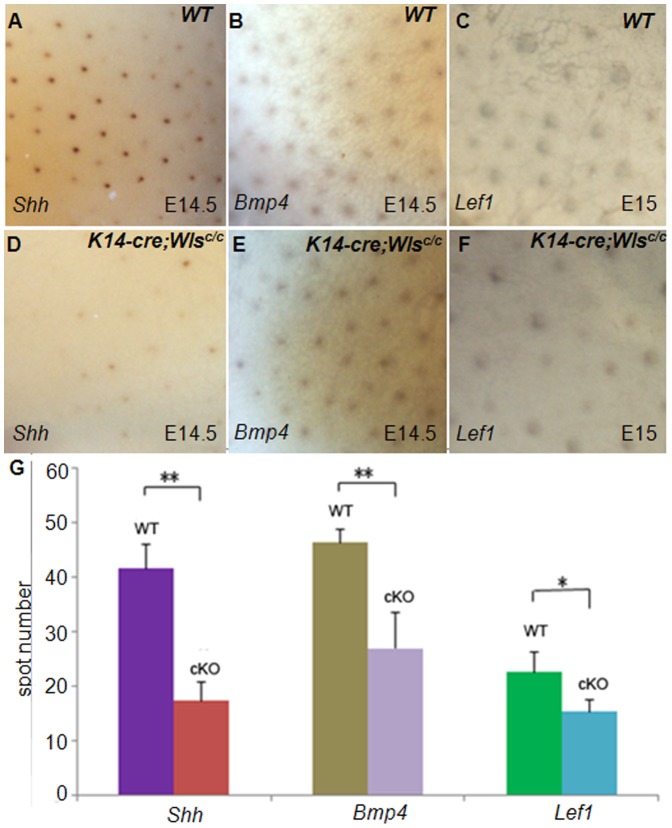
Hair follicle placode formation is impaired without Wls. (A and D) *Shh* positive placodes are decreased in *Wls*-deficient skin compared with the control. (B and E) *Bmp4* expressing dermal condensates are reduced in *K14-cre;Wls^c/c^* mutants. (C and F) *Lef1* positive placodes decrease significantly in *Wls*-deficient skin. (G) Statistical analysis of the *Shh*, *Bmp4* and *Lef1* positive spots in embryo skin. **, P<0.01; *, P<0.05. cKO, *K14-cre;Wls^c/c^*.

### Histology, Immunohistochemistry (IHC), and X-gal Staining

Skin tissues were harvested at specific stages and fixed with 4% PFA in PBS. The fixed samples were dehydrated, embedded with paraffin, and cut into 10 µm thick sections. H&E staining was performed on rehydrated samples. For IHC, sections were rehydrated, blocked with 5% goat serum, and incubated overnight at 4°C with primary antibodies including anti-Wls (Santa Cruz, USA), anti-β-catenin (CST, USA), anti-Lef1 (CST, USA), and anti-Wnt5a (R&D system, USA) according to the recommended ratio. Alexa Fluor® 488 (Invitrogen, USA) was used as the second antibody. The labeled samples were then counterstained with DAPI, and a Leica confocal microscope was used to observe and document the results. Whole mount X-gal staining was performed as previously described [17]. After re-fixing the stained samples, they were either photographed or sectioned to observe the Cre enzyme activity.

### In Situ Hybridization, Quantification and Statistical Analysis

Mice were maintained on a 12 h light/dark cycle, and females with vaginal plugs at 12 a.m. were designated 0.5 d.p.c. After sacrificing the mice at specific stages, embryos were collected and fixed with 4% PFA overnight at 4°C. The in situ hybridizations of *Bmp4*, *Shh*, and *Lef1* were conducted according to Zhu et al. [12]. The signal-positive spots of each probe in mutant skin were counted from three different regions of 2X2 mm square, and compared with the counterpart regions of their normal littermates. Statistical significance was measured by Student’s *t*-test.

**Figure 4:**
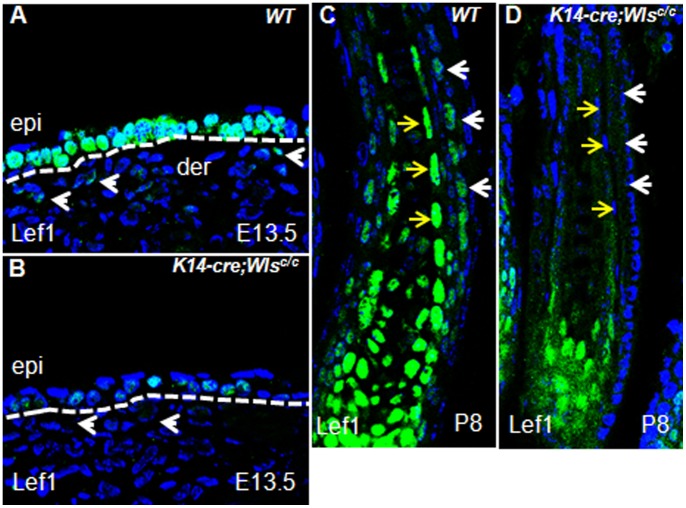
Wnt/β-catenin pathway is impaired in *Wls*-deficient skin. (A and B) Lef1 expression in the surface ectoderm is decreased, and no signal is detected in the underlying dermal mesenchyme (white arrows) in *Wls*-deficient skin. (C and D) Lef1 is detected both in ORS (white arrows) and IRS (yellow arrows) of control hair follicles. However the expression is indistinguishable in hair follicles of *K14-cre;Wls^c/c^* skin. Abbreviations: epi, epidermis; der, dermis.

## Results

### *Wls* is Expressed Dynamically during Hair Follicle Induction and Hair Cycling

Although *Wls* is reportedly expressed in hair follicles [18], its expression during hair follicle induction and hair cycling is still unknown. Given that Wnt signaling is required for the induction of placodes, we first examined *Wls* expression in E13.5 upon the initiation of placode formation. As expected, Wls was uniformly expressed on surface ectoderm and the underlying dermal mesenchyme in dorsal skin (Fig. 1A). At E14.5 when nascent placodes were clearly formed, Wls protein was detected in the epidermis and placodes (Fig. 1B). Parallel with the growth of placodes, Wls expression persisted in the placodes and epidermis at E15.5 (Fig. 1C). These results indicate the potential requirement of *Wls* for hair follicle induction and growth. We then investigated the *Wls* expression pattern during hair cycling after birth. In early anagen, strong *Wls* expression was detected in the bulge region, together with dermal papilla. Wls expression was also observed in the second hair germ, which connects the bulge and dermal papilla (Fig. 1D). When the hair follicles grew and reached the full anagen stage, *Wls* was strongly expressed in the keratinocyte matrix and pre-hair shaft region. In contrast, expression in dermal papilla was weak and there was no obvious signal in the dermal sheath (Fig. 1E). In the early catagen stage when hair follicles stop growing and begin to regress, Wls expression persisted in the inner root sheath (IRS), outer root sheath (ORS), and hair shaft, but remained weak in dermal papilla (Fig. 1F). However, in mid-catagen, *Wls* expression began to increase in dermal papilla but decreased in other hair follicle compartments. In the epithelial strand where strong apoptosis persists [19], *Wls* expression was almost absent (Fig. 1 G). In the late catagen stage, Wls was mainly detected in the lower part of hair follicles including dermal papillae (Fig. 1H). In the telogen stage, Wls was mainly expressed in the bulge region and the expression in dermal papilla was relatively weak (Fig. 1I). The dynamic expression pattern of *Wls* strongly indicates that it may regulate hair follicle development and hair cycling.

**Figure 5:**
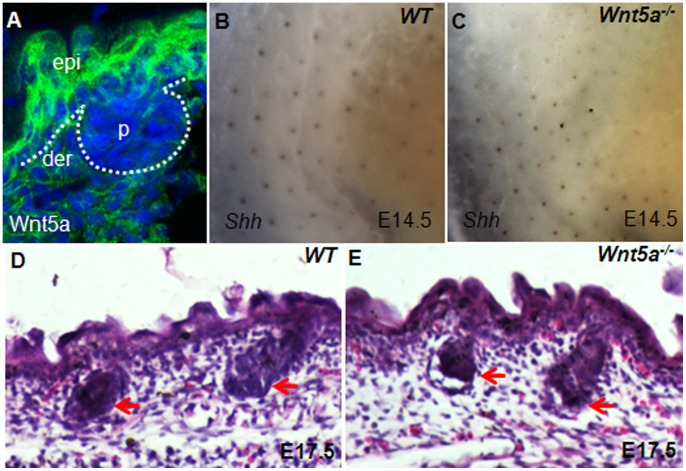
Wnt5a is expressed in the embryonic epidermis but not required for hair follicle induction. (A) Wnt5a highly expressed in the epidermis, and its expression in the dermis is relatively weak, no significant expression is detected in developing placodes. (B and C) *Shh* is normally expressed in placodes in Wnt5a null embryos at E14.5. (D and E) The down-growth of embryonic hair follicles in *Wnt5a*-deficient skin is unaffected, as indicated by red arrows. Abbreviations: epi, epidermis; p, placode.

### *K14-cre;Wls^c/c^* Mice Display Significant Hair Loss

To determine whether Wls is necessary for hair follicle development, we specifically deleted *Wls* with K14-cre, which has strong activity in the basal layer of the epidermis and outer root sheath of hair follicles [5]. *K14-cre;Wls^c/c^* mice and their control littermates were born normally, viable, and fertile, however, their skin had patched hair loss (Fig. 2A). This phenotype showed similarity with that of *K14-cre;β-catenin^c/c^* mice (Fig. 2B). The hair loss phenotype varied among individuals and usually did not have fixed regions. However, almost all hairs were lost within one month after birth (data not shown). By sectioning dorsal skin of *K14-cre;Wls^c/c^* mice and comparing with the control, we found that the hair follicles almost disappeared in the hairless patches (black arrow in Fig. 2 D). To explain the patched hair loss phenotype, we re-evaluated K14-cre activity of this transgenic mice line in the skin and found ubiquitous cre activity in placodes at E15.5 (Fig. 2E): cre activity was strongly presented in the inter-placode epidermal basal layer within some patches, but was weak in other regions (Fig. 2F and 2G). Therefore, the different K14-cre activity may be responsible for the phenotype of “patched” instead of complete hair loss. We then examined the Wls protein in mutant skin to evaluate the efficiency of Wls deletion. Compared with the control, Wls protein was almost disappeared in the surface ectoderm at E13.5 and in the epidermis of the hairless patches after birth (Fig. 2H–2K).

### Embryonic Hair Follicle Induction is Impaired without Epidermal Wls

Decreased hair follicle number may result from defective hair follicle induction. Thus, we next analyzed mutant skin at the molecular level. *Shh* is expressed in placodes whereas *Bmp4* is expressed in the underlying mesenchymal condensates in wild-type mice, and both genes are required for proper hair development and serve as good markers of developing placodes [5], [20], [21]. In *Wls*-deficient skin, both *Shh* and *Bmp4* positive spots were significantly reduced (Fig. 3A, 3B, 3D, 3E and 3G). Lef1 is another marker for placodes, and it is also a direct target of Wnt/β-catenin pathway [5]. Consistently, we found reduced *Lef1* positive spots in Wls-deleted skin (Fig. 3C, 3F and 3G). Overall, these results indicate that Wls is required for the proper induction of hair follicles.

### Deletion of Wls Disrupts the Wnt/β-catenin Pathway

As aforementioned, the defects of Wls mutants are similar with those of β-catenin mutants, thus, *Wls* deletion may affect the Wnt/β-catenin pathway. Consequently, we examined Lef1 protein in *Wls*-deficient skin by IHC at E13.5. In *K14-cre;Wls^c/c^* skin, Lef1 expression decreased in the surface ectoderm and was undetectable in the underlying mesenchyme (Fig. 4A and 4B). We then performed IHC on postnatal hairless skin to evaluate the Lef1 level. As expected, Lef1 expression in ORS and IRS was absent in *Wls*-deleted hair follicles (Fig. 4C and 4D). These results suggest that impaired Wnt/β-catenin pathway may be responsible for the defects in *Wls*-deficient skin.

### Wnt5a is Expressed in the Epidermis but not Required for Hair Follicle Induction

Wnt5a is another important Wnt ligand that plays essential roles in many developmental processes [22]. Its functions in hair follicle morphogenesis and differentiation are only begining to be revealed and not yet fully recognized [8]. Considering that Wls also mediates Wnt5a secretion, we cannot exclude the possibility that Wnt5a pathway impairment may contribute to the defect in hair follicle induction in *K14-cre;Wls^c/c^* skin. Therefore, we investigated whether Wnt5a plays such a role in regulating hair follicle induction. The IHC results revealed that Wnt5a was expressed both in the epidermis and dermis, but the expression level in epidermis seems higher than that in dermis. Wnt5a expression in developing placodes was obviously weaker than in surrounding tissues (Fig. 5A). We then investigated hair follicle induction in *Wnt5a* null skin. At E14.5, there was normal *Shh* expression in *Wnt5a* null skin, indicating correct placode formation (Fig. 5B and 5C). The subsequent down-growth of hair follicles was also neither obviously affected at E17.5 without *Wnt5a* (Fig. 5D and 5E). Thus, Wnt5a is dispensable for hair follicle induction and disruption of Wnt5a signaling in epidermis may not contribute to the defects of Wls deficient skin.

## Discussion

The importance of *Wls* gene in multiple developmental processes has been revealed in recent years, and its requirement for Wnt secretion is conserved among species [9], [12], [23]. Evidence from both cell lines and mouse genetic studies confirm that Wls is required for the secretion of various Wnts, including canonical and non-canonical Wnts, represented by Wnt3 and Wnt5a, respectively [11], [12]. By manipulating the *Wls* gene, we can overcome the redundancy of Wnts in certain tissues and organs, and thus determine Wnt function in detail. Wnt signaling in the skin regulates hair follicle induction and hair cycling at different stages. Considering that many Wnts are expressed during embryonic hair follicle induction and morphogenesis, as well as the possible functional redundancy of these Wnts, using *Wls*-modified mice in hair follicle research is promising.

The expression of *Wl*s during hair follicle initiation and hair cycling was studied in this study. We found uniform Wls expression in embryonic epidermis from E13.5 to E15.5, and in developing placodes. During the preparation of the manuscript, Chen and colleagues also found Wls expression in embryonic skin [24]. After hair morphogenesis completion, almost every hair follicle compartment displayed Wls distribution. Interestingly, the expression of Wls usually overlaps with those of various Wnt ligands. For example, Wls is highly expressed in embryonic epidermis and placodes, and this pattern overlaps with those of canonical Wnt3, Wnt6 and Wnt10b ligands [7]. In early anagen, Wls protein strongly presents in the bulge region and second hair germ, where Wnt10a and Wnt10b expression are up-regulated [7]. In full anagen, Wls was strongly expressed in ORS, IRS, pre-hair shaft, and matrix. Correspondingly, non-canonical Wnt5a, canonical Wnt3a, Wnt3, and Wnt10b are expressed in these compartments, respectively [7]. Moreover, Wls seemed to be preferentially expressed overlapping with canonical Wnts. This finding is reasonable because *Wls* itself is a direct target of the Wnt/β-catenin pathway [11]. In the catagen stage, Wls expression began to regress in ORS, IRS, and hair shaft, but expression in dermal papilla progressively increased. In telogen, bulge Wls expression was maintained, although at a low level. The biological meaning of this expression needs further investigation.

By depleting *Wls* using K14-cre, we found impaired hair follicle induction in Wls-deficient skin. This phenotype was similar to that of β-catenin knockout by the same cre (Fig. 2). Indeed, the activity of Wnt/β-catenin in *Wls*-deficient skin was significantly impaired, as revealed by Lef1 expression (Fig. 4). Meanwhile, we also found strong expression of Wnt5a in wild type embryonic epidermis. As Wls is also essential for Wnt5a secretion, the defects in *Wls* mutant skin could also due to impairment of Wnt5a pathway. However, our analysis of Wnt5a null embryo indicates there is no obvious defect of hair follicle induction in *Wnt5a* deficient skin (Fig. 5). Thus, Wnt5a seems not to be necessary for hair follicle induction. Taken together, our results suggest that Wls is required for hair follicle induction, possibly by mediating the Wnt/β-catenin pathway.
